# Cu^∥^-loaded polydopamine coatings with *in situ* nitric oxide generation function for improved hemocompatibility

**DOI:** 10.1093/rb/rbz043

**Published:** 2020-01-17

**Authors:** Lei Zhou, Xin Li, Kebing Wang, Fangyu Shen, Lu Zhang, Peichuang Li, Tengda Shang, Jin Wang, Nan Huang

**Affiliations:** Key Laboratories of Advanced Technology for Materials of Education Ministry, School of Materials Science and Engineering, Southwest Jiaotong University, Chengdu, Sichuan 610031, China

**Keywords:** nitric oxide, polydopamine coating, copper ion, hemocompatibility

## Abstract

NO is the earliest discovered gas signal molecule which is produced by normal healthy endothelial cells, and it has many functions, such as maintaining cardiovascular homeostasis, regulating vasodilation, inhibiting intimal hyperplasia and preventing atherosclerosis in the blood system. Insufficient NO release is often observed in the pathological environment, for instance atherosclerosis. It was discovered that NO could be released from the human endogenous NO donor by many compounds, and these methods can be used for the treatment of certain diseases in the blood system. In this work, a series of copper-loaded polydopamine (PDA) coatings were produced through self-polymerization time for 24, 48 and 72 h. The chemical composition and structure, coating thickness and hydrophilicity of the different copper-loaded PDA coatings surfaces were characterized by phenol hydroxyl quantitative, X-ray photoelectron spectroscopy, ellipsometry atomic force microscopy and water contact angles. The results indicate that the thickness and the surface phenolic hydroxyl density of the PDA coatings increased with the polymerization time.This copper-loaded coating has glutathione peroxidase-like activity, and it has the capability of catalyzing NO releasing from GSNO. The surface of the coating showed desirable hemocompatibility, the adhesion and activation of platelets were inhibited on the copper-loaded coatings. At the same time, the formation of the thrombosis was also suppressed. These copper-loaded PDA coatings could provide a promising platform for the development of blood contact materials.

## Introduction

NO was generally considered to be one of the atmospheric pollutants, and it was regarded as one toxic and harmful gas before 1987. NO was not proved to be endothelium-derived derivative factor until it was discovered in the vasculature by Robert Furchgott, Louis Ignarro and Ferid Murad [1], and the research on NO began a new chapter. With the continuous research and development, it was demonstrated that NO possessed variety of physiological and pathological functions such as cardiovascular homeostasis [2], wound repair [3], tumor behavior [4] and immune response [5]. In 1992, it was named ‘Molecular of the Year’ by the *Science* Magazine [6], and numerous NO-based treatment strategies have emerged, including treatment of cardiovascular disease (CVD) [7], wound healing [8], antibacterial effects [9] and cancer treatment [10].

NO is produced by endothelial nitric oxide synthase (eNOS) catalyzed oxidation of l-arginine in vascular endothelial cells, and it has pleiotropic effects including maintaining cardiovascular homeostasis, regulating vasodilation, promoting ECs growth, maintaining ECs integrity, inhibiting platelet and leukocyte adhesion, aggregation, inhibiting SMCs proliferation and preventing atherosclerosis [11–13]. The adequately and continuously releasing of NO is an important indicator for healthy EC. NO also plays an important role in regulating the mitosis and proliferation of SMC and inhibiting the adhesion and aggregation of platelets [14]. Due to the various biological effects of NO in the vascular system, researchers have developed a number of blood contact devices based on NO treatment strategies, such as artificial blood vessels [15, 16], biosensor [17], vascular stents [7, 18], etc. The strategies for constructing NO-generating medical devices could be divided into two methods: NO release methods and catalytic NO generation method [19]. For the NO release method, there are mainly two ways. The first way is to directly load the NO molecule on the materials. Cavalieri [20] encloses the gaseous NO molecule with poly(vinyl alcohol) microbubbles or utilizes the zeolite and the metal organic skeleton [7, 21, 22]. The porous material adsorbs gaseous NO in the porous network. The second approach is loading NO donors into the coatings or nanostructures of the materials [23–25], such as S-nitrosothiols (RSNO) or N-diazeniumdiolates (NONOates). However, the NO release method, whether using NO gas molecules or NO donors, has limited storage of NO [26]. The NO release amount and duration are difficult to control and regulate, which is not conducive for long-term implanted of the medical devices. For the catalytic NO-generating method, the NO donor is derived from the body, such as S-nitrosoglutathione (GSNO), S-nitroso albumin (AlbNO) and S-nitrosocysteine (CysNO) [27–29]. The production of NO can dynamically regulate the sustained release of NO in response to changes in the microenvironment [30].

A variety of substances have been reported that they are able to catalyze endogenous NO donor releasing NO, such as diselenodipropionic acid, cystamine and selenocyst-amine [30–32]. They have glutathione peroxidase (GPx)-like activity and catalyze NO releasing from donors *in situ*. Copper is a trace element required by the human body and it is widely distributed in the body [33], which participant many important life processes. It has the potential to catalyze the NO releasing from RSNO in the blood [34, 35]. Since NO is a gas signal molecule with multiple effects, it possesses various biological functions in the blood system [11–13, 36]. Therefore, exploring a coating that can catalyze NO release from diseased region may provide a new direction for the development of blood contact materials.

This study proposes a strategy for building NO catalyzing releasing coatings via loading copper ions into a PDA coatings. Dopamine has been shown to be coated on many substrate surfaces [37]. It has a strong ability to coordinate copper ions due to plenty of catechol groups. The building copper-loaded (PDA) has advantages of simple operation, mild conditions and no material or shape limitation [38]. In this study, we explored the ability of the copper-loaded coating to catalyze the release of NO, and the hemocompatibility of the copper-loaded coatings were also evaluated.

## Materials and methods

### Materials

Copper chloride (CuCl_2_) was purchased from Aladdin Reagent. Dopamine and Tris base were supplied from Sigma-Aldrich. All other chemical agents were analytical grade and used without further treatment.

### Preparation of copper-loaded PDA coating

The copper-loaded PDA coatings were fabricated by immersing the substrate materials in different solutions. Specifically speaking, a 0.5mg/ml dopamine solution (pH = 8.5, 1.2 mg/ml Tris base buffer) and an aqueous solution of 2 mg/ml copper chloride (DI water) were advance prepared. Then, the substrates, including 316L stainless steel (SS), silicon (Si) wafer or titanium (Ti), were dipped in dopamine solution, and then washed by DI water after immersed 24, 48 and 72 h, respectively. The samples were named PDA24, PDA48 and PDA72. Then, the samples were immersed in an aqueous solution of 2 mg/ml CuCl_2_. At last, the samples were washed with DI water after soaking 3 h. The immersed samples were named PDA24Cu3, PDA48Cu3, PDA72Cu3, respectively.

### Characterization of copper-loaded coatings

The thickness of the different samples was measured by an ellipsometer (EP3SW, Accurion Co.) at a fixed angle of incidence (60°) and wavelength (658 nm). All coating thickness data were obtained by the Cauchy mode. The surface topography and roughness of samples were measured by an atomic force microscope (AFM SPA400, SEIKO, Japan). In this experiment, AFM was used to detect the surface roughness of PDA24, PDA48, PDA72, PDA24 Cu3, PDA48 Cu3 and PDA72 Cu3 coatings which were deposited on the silicon wafers. The contact angle apparatus (DSA 100, Krüss, GmbH, Germany) was used to evaluate the change of the hydrophilicity and hydrophobicity of the surfaces the samples. All tests were performed using DI water at room temperature. In order to investigate the copper ion carrying ability of the different PDA coatings, the surface density of phenolic hydroxyl groups was tested. The Micro-BCA method was used to evaluate the surface phenolic hydroxyl groups density of each 316 L SS. The phenolic hydroxyl group can reduce Cu^2+^ to Cu^+^ under alkaline conditions, and the Cu^+^ can react with bicinchoninic acid (BCA) forming colored complex. The complex is purple and exhibits strong absorbance at 562 nm [39]. The X-ray photoelectron spectroscopy (XPS, XSAM800, Kratos Ltd, UK) was used to measure the surface chemical compositions of the samples. All XPS data were obtained with an Al Kα as the gun source and a take-off angle of 90°.

### Measurement of NO generation

A chemiluminescence NO analyzer (NOA) (Seivers 280i, Boulder, CO) was used to monitor the real time release of NO. Here, copper-loaded PDA coatings and copper-free samples were prepared on Ti foil (0.2 × 1 cm). The samples were immersed into a test solution containing SNAP (10 μM) and glutathione (GSH, 10 μM). NO produced by membrane catalysis was delivered to the NO analyzer through a stream of N_2_ (g).

### Copper ion releasing test

Micro-BCA can be used to detect the phenolic hydroxyl groups on the surface of the material. Based on this principle, the copper content on the surface also can be measured using the Micro-BCA assay. Briefly, GSH solution could react with Cu^2+^to form Cu^+^. Then BCA alkaline solution could react with Cu^+^ and form colored complex which have the maximum absorption at 562 nm. Then, a standard curve was prepared at 562 nm using a soluble copper salt. The absorbance of the purple complex at 562 nm on different samples was examined, and the copper ion release rate was calculated by comparing the standard curve.

### *Semi-in vivo* whole blood dynamic circulation experiment

In order to evaluate the hemocompatibility of copper-loaded coating, a semi-*in vivo* whole blood dynamic circulation system was established. Briefly, the system consisted of a New Zealand White Rabbit and one pipeline, the end of which connected the rabbit’s carotid artery and jugular vein. A sample of the copper-loaded coating prepared on the 316L stainless steel foil was placed in the pipeline before the start of the experiment. After circulating the blood for 1 h, the foil was taken out and washed with PBS for three times. Then the surface of the foil was capture by using a digital camera. The foil was fixed with glutaraldehyde aqueous (2.5%) for 4 h. The sample was then dehydrated, de-alcoholate and gold sputtered. The morphology of coating was observed using SEM (Quanta 200, FEI, Holland).

### Statistical analysis

All quantitative analysis experiments were repeated at least three times independently for statistical significance. The results of data were expressed as mean ± standard deviation (SD). Data analysis was carried out by one-way analysis of variance (ANOVA). The *P* values were regarded as statistically significant when the probability value of less than 0.05.

## Results and discussion

### Analysis of film thickness detection results of ellipsometry

**Figure 1:**
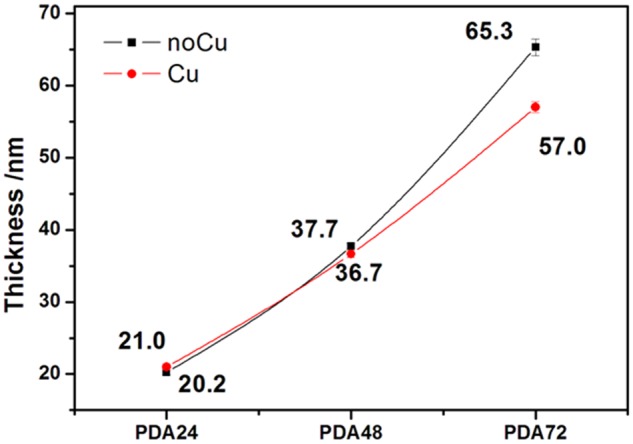
Thickness of polydopamine coatings (with or without copper ion loaded)

The coatings were prepared on Si wafer and the film thickness of the PDA coatings was measured by an ellipsometer, before and after copper ion loaded. The PDA coatings were deposited on the silicon wafers surfaces. As shown in Fig. 1, with the deposition time increasing, the film thickness of the coatings increased. The PDA coating deposition rate between 48  and 72 h increased compared with the first 48 h. Since the PDA coatings undergo the soaking treatment with the CuCl_2_ solution, the thickness of the copper-loaded PDA coatings is not significantly different from the thickness of the unloaded copper.

### Surface topography and roughness

**Figure 2:**
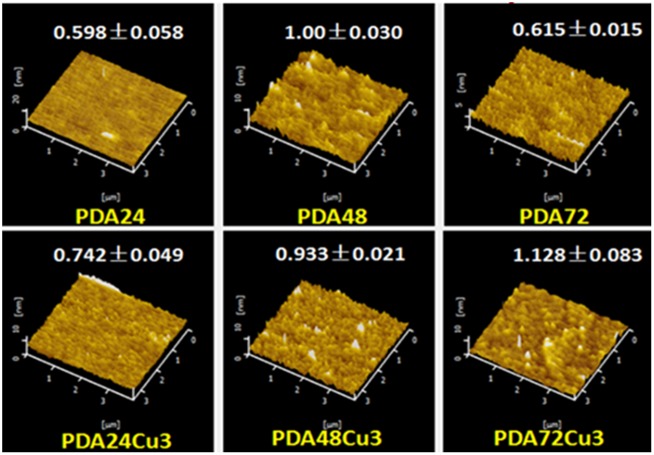
Surface topography and roughness of different samples (roughness unit nm)

Surface topography and roughness calculations were performed on several process coatings deposited on the surface of Si wafers by AFM. As shown in Fig. 2, it was observed that the surface of several films was relatively flat, and the surface roughness of the coatings was between 0.6 and 1.2 nm. The samples with less deposition time have smaller surface roughness. 3D image also shows that the surface of the PDA24 sample was the flattest. For the copper-loaded samples, the surface roughness of each samples showed an upward trend. However, the surface roughness of the PDA coatings did not have obvious difference, whether copper ion loaded or not. The surface topography of copper-load and copper-free coatings did not change regularly.

### Quantitative phenolic hydroxylation of surface

**Figure 3:**
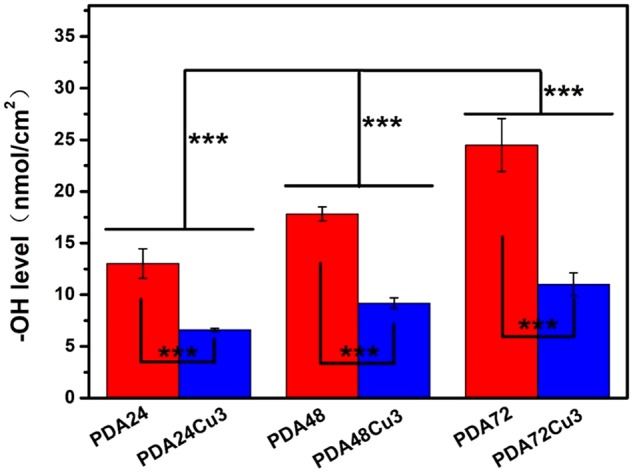
Surface Ar-OH density of the copper-free and copper-loaded samples

The results of Micro-BCA assay for detecting phenolic hydroxyl density on each 316L SS surface are shown in Fig. 3. For the copper-free samples, the surface phenolic hydroxyl density increased with the deposition time, which indicated that the surface phenolic groups augmented over time. The previous film thickness tested by the ellipsometry confirmed that the film thickness increased with the reaction time.

There would be mutual penetration between different layers, resulting in a growing density of functional groups on the surface of coatings. For the copper-loaded samples compared with copper-free samples, the density of phenolic groups was significantly reduced. In other words, the loading of copper consumes a part of phenolic groups. Comparing the three groups of samples, the surface phenolic hydroxyl density of the sample decreased by nearly 50% after loading copper ions.

### Surface hydrophobicity

**Figure 4:**
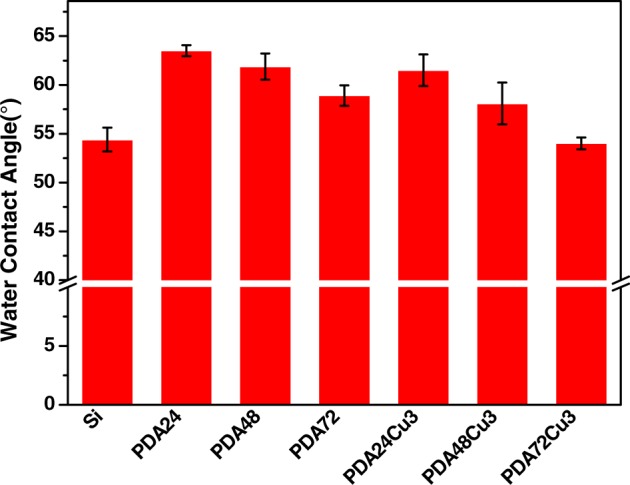
The WCA of different samples

The water contact angle (WCA) of samples was shown in Fig. 4. The WCAs of the PDA samples surfaces were increased compared with Si. As the dopamine deposition time increased, the WCA of the samples decreased. Copper-loaded and copper-free samples exhibited similar trends. The sample with the largest WCA was PDA24Cu3, which was 61.5°. The PDA deposition time was longer, the WCA was smaller. The PDA coating had many specific functional groups, such as benzene, quinone, phenolic hydroxyl group and amino group. The phenolic hydroxyl group and amino group could increase the surface hydrophilcity. With the increase of dopamine deposition time, the phenolic hydroxyl group content of surface and hydrophilicity were increased. The WCA of sample was gradually decreased. For the same deposition time, the WCA of sample had a reduction of nearly 3° after loading copper. The surface functional groups were changed after loading copper possibly because of combination copper with phenolic hydroxyl group, which made hydrophilicity changed.

### XPS testing

**Figure 5:**
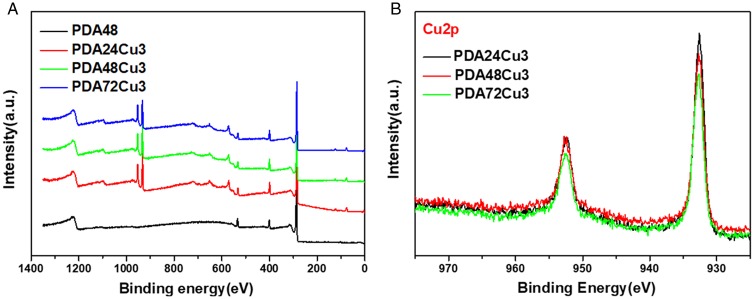
(**A**) XPS wide scans of different samples. (**B**) High resolution of copper-loaded samples

**Figure 6:**
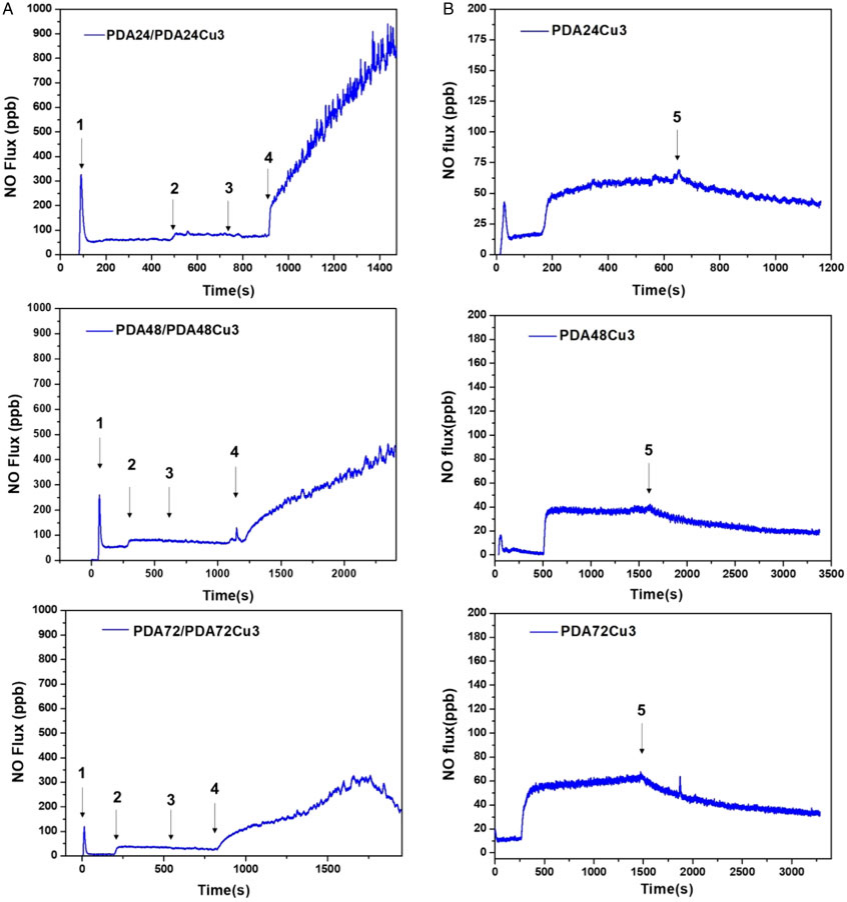
(**A**) NO-generating rate of different samples (1 indicates the addition of donor SNAP, 2 indicates the addition of copper-free samples, 3 indicates removal of copper-free samples, and 4 indicates the addition of copper-loaded samples). (**B**) NO-generating rate of different samples after immersed for 6 h (5 indicates removal of samples)

XPS test was performed to observe the chemical structure and composition of the copper-loaded and copper-free coatings on 316L SS. Since the elemental composition of the PDA24, PDA48, PDA72 have no significant differences, so the PDA48 sample was chosen as the PDA coating before copper loaded in this test. As shown in Fig. 5A, there were three peaks at the binding energy of 284.3, 399.0 and 532.0 eV appearing in the full spectrum data of PDA48, PDA24Cu3, PDA48Cu3 and PDA72Cu3. These three peaks were belong to the peak of C1s, N1s and O1s, respectively. At 932 and 952 eV, the copper -loaded sample showed two peaks which belonged to the peaks of Cu2p1/2 and Cu2p3/2. The peaks of 568.1, 635.0, 647.5 and719.5 eV were the Auger electron characteristic peak of Cu. The Cu high-resolution XPS was also carried out. As shown in Fig. 6, there were peaks displayed at 932 eV and 952 eV of the copper-loaded samples, respectively. The shape and position of peaks for copper elements did not show significant differences for the copper-loaded surface of three different deposition times. From Fig. 5B, no peak was observed between 938 and 945 eV, indicating that the surface did not contain Cu^2+^. Because if there is Cu^2+^ on the surface, a satellite peak appears between 938 and 945 eV [40].

### NO generation from copper-loaded coatings

The NO release rate was measured by chemiluminescence method using NOA280i instruments. As shown in Fig. 6A, the NO release rate rapidly increased after the supplement of the S-nitroso-N-acetylpenicillamine (SNAP). Then the rate decreased between 0 and 50 ppb. This NO releasing was considered to be the self-decomposition of SNAP. Subsequently, the copper-free sample was immersed into this reaction solution, and it could be seen that the PDA coating sample can increase the NO release rate by 30–50 ppb. There was no significant change after the sample was removed. Then, adding the copper-loaded samples, the NO releasing rate significantly increased. The PDA24Cu3 sample was added to the reaction solution. The NO production rate rose linearly within 500 s, and NO releasing rate increased from the initial about 100–900 ppb. However, it dropped to 0 after 1400 s, due to the consumption of the donor in the reaction chamber. The NO releasing rate was raised from the initial 100 to 280 ppb in 500 s after the PDA48Cu3 sample was inserted into the reaction solution. In the later period, it also showed steady growth. After the PDA72Cu3 sample was inserted into the reaction solution, there showed the similar NO release behavior to PDA48Cu3. However, the releasing rate of NO produced by the PDA72Cu3 sample increased from about 30 ppb to about 170 ppb within the first 500 s, which was smaller than that of the previous two samples. It was may be due to the samples with long-term dopamine deposition had denser phenolic hydroxyl functional groups and had stronger chelation to copper ions. In a short period of time, the surface exposed or released copper ions were less than the PDA24Cu3, PDA48Cu3 samples. So the production rate of NO was lower. In order to investigate the catalytic stability of the copper-loaded coatings, the samples were immersed into the reaction solution for 6 h. As shown in Fig. 6B, the samples which immersed for 6 h were added to the reaction solution, and the NO production rates was stable compared with the un-immersed samples. The NO release rate of the PDA24Cu3 was approximately between 50 and 75 ppb. Its release rate was significantly reduced and stable compared to that before immersion. After the sample was removed at 650 s, NO release was still visible, but the release was slowly reduced. Similarly, a same phenomenon occurred in the PDA48Cu3 and PDA72Cu3.The NO release rates of the two samples were about 40 ppb and 50 ppb, respectively. After the sample was removed, NO release was also observed, but the release was slowly reduced. The reason why the sample can steadily catalyze the decrease of NO release rate after soaking may be that the bound unstable copper ions had been released and the copper ions firmly bound by the phenolic hydroxyl group can stably catalyze the release of NO by RSNO during the soaking process. The coating still contained a small amount of unstable copper ions after the sample was immersed for 6 h, so a small amount of NO was released.

### Short-time soaking release of copper from coatings

**Figure 7:**
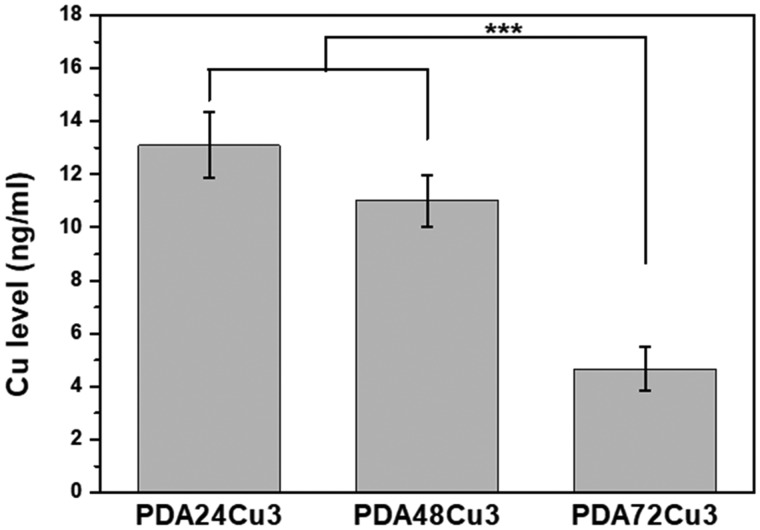
The Cu releasing amount from the surface of the copper-loaded samples by 1 h immersion

Figure 7 shows the amount of Cu released from the surface of the copper-loaded 316L SS using the Micro-BCA kit. The copper releasing amount from the surfaces of PDA24Cu3, PDA48Cu3, PDA72Cu3 samples was 13.09 ± 1.25, 11.02 ± 0.97, 4.65 ± 0.83 ng/ml, respectively, after immersed in the test solution at 37°C for 1 h. Comparing the samples with different dopamine reaction times, it was found that the longer the dopamine deposition time, the less the releasing amount of copper ions. Comparing the data of the NO production, the results of the surface phenolic hydroxyl groups and the data of thickness of the coatings, it can be speculated that the more the surface phenolic hydroxyl functional group and the thicker the coating, the more stable copper loading and the less copper releasing.

### Hemocompatibility

**Figure 8:**
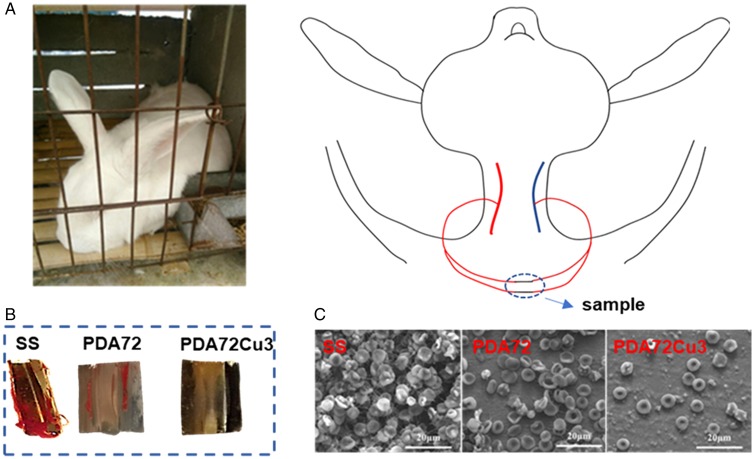
(**A**) Schematic diagram of the semi-*in vivo* whole blood dynamic circulation system for hemocompatibility assessment; (**B**) digital photos of SS, PDA72, PDA72Cu3; and (**C**) SEM photos of SS, PDA72, PDA72Cu3

The anticoagulant effect of blood contact materials is critical for thrombosis. Therefore, the anticoagulant behavior of the copper-loaded coating in the blood was further investigated. For blood compatibility assessment, a semi-*in vivo* whole blood dynamic circulation system was established, which consisted of New Zealand Whiter Rabbits and one pipe, as shown in Fig. 8A. The sample was placed in the tube and exposed to blood for 1 h. It could be seen that the surface of the 316L stainless steel was covered a large number of blood clots, and the PDA72 sample was covered a small amount of blood clots, while no obvious clots were observed on the surface of the PDA72Cu3 sample, as shown in Fig. 8B. It could be seen from the SEM photograph that no substrate was observed on the surface of the SS, and it was composed of a large amount of fibrinogen network and fibrin-packed red blood cells (RBC). There was also a thrombus on the surface of the PDA72 sample, but the thrombus was significantly lower than the SS and did not completely cover the surface of the substrate. Due to the thrombus composed of fibrin and RBCs, most of the RBCs become acanthocytes, which appeared on both SS and PDA72 samples. However, only few RBCs were observed on the surface of PDA72Cu3, and no thrombus or fibrinogen network was observed. In a word, copper-loaded coatings had better hemocompatibility than SS and PDA. Due to RSNO is abundant in the blood and can be catalyzed and decomposed by copper ions to release NO that has anticoagulation and inhibits platelet activation. Therefore, the copper-loaded coating can continuously catalyze endogenous RSNO to produce NO, and the NO can continue to inhibit platelet adhesion, activation and aggregation.

## Conclusion

The core problem of long-term implanted blood contact materials is how to achieve anticoagulant effects based on the functionality of the material. NO, which can inhibit platelet adhesion and activation, is a signal molecule in the body. The abundant RSNO in the body can be catalytically decomposed by Cu^+^. This process can release NO. This study proposes a surface modification strategy based on PDA coating-mediated copper immobilization to address the complications associated with implanted blood contact materials. The method was simple and convenient, and had no shape limitations. The modified surface was capable of catalyzing RSNO decomposition and NO release. In the PDA coating, copper ions were deeply buried inside the coating structure, and as a result, the coating can continuously generate NO, thereby ensuring antithrombotic for a long period of time. At the same time, the NO release rate was a good inhibitor of thrombus formation. Overall, our work provides a surface modification method and a promising coating for blood contact materials.

## Acknowledgements 

This work was financially supported by the National Key Research and Development Program of China (2017YFB0702504), the National Natural Science Foundation of China (NSFC Project 81801853), the Postdoctoral Science Foundation of China (2018M633400) and the Sichuan Science and Technology Program (19GJHZ0058). 

*Conflict of interest statement*. None declared.
